# SLC31A1 Identifying a Novel Biomarker with Potential Prognostic and Immunotherapeutic Potential in Pan-Cancer

**DOI:** 10.3390/biomedicines11112884

**Published:** 2023-10-25

**Authors:** Pei Zhang, Heqi Yang, Kaiguo Zhu, Chen Chang, Wanrui Lv, Ruizhen Li, Xiaoying Li, Tinghong Ye, Dan Cao

**Affiliations:** 1 Division of Abdominal Tumor Multimodality Treatment, Cancer Center, West China Hospital, Sichuan University, No. 37 Guoxue Alley, Chengdu 610041, China; medzpei@163.com (P.Z.); yang-heqi-scu@outlook.com (H.Y.); 15680790262@163.com (K.Z.); changch1008@163.com (C.C.); 18990603639@163.com (W.L.); liruizhendoctor@163.com (R.L.); lxy25464@163.com (X.L.); 2 Sichuan University-Oxford University Huaxi Gastrointestinal Cancer Centre, State Key Laboratory of Biotherapy, West China Hospital, Sichuan University, Chengdu 610041, China; yeth1309@scu.edu.cn

**Keywords:** cuproptosis, *SLC31A1*, immunity, prognosis, pan-cancer

## Abstract

Solute carrier family 31 member 1 (*SLC31A1*) encodes a protein that functions as a homotrimer for the uptake of dietary copper. As a vital member of the cuproptosis gene family, it plays an essential role in both normal tissues and tumors. In this study, we analyzed *SLC31A1* across human cancer types to gain a better understanding of *SLC31A1*’s role in cancer development. We searched for information using online databases to analyze, systematically and comprehensively, the role of *SLC31A1* in tumors. Amongst nine cancer types, the expression of *SLC31A1* was significantly different between tumors and normal tissues. According to further analysis, pancreatic cancer had the highest mutation rate of the *SLC31A1* gene, and the methylation levels of the gene were significantly reduced in seven tumors. The expression of *SLC31A1* is also linked to the infiltration of tumors by immune cells, the expression of immune checkpoint genes, and immunotherapy markers (TMB and MSI), suggesting that *SLC31A1* may be of particular relevance in immunotherapy. This thorough analysis of *SLC31A1* across different types of cancer gives us a clear and comprehensive insight into its role in causing cancer on a systemic level.

## 1. Introduction

Despite advances in treatment and prevention, cancer still ranks second among the leading causes of death worldwide [1]. GLOBOCAN estimates that, in 2040, cancer cases and deaths will have risen to 27.5 million new cases and 16.3 million deaths, making it one of the leading causes of death worldwide [2]. Types of cancer and patients are characterized according to biological heterogeneity, according to genomic and epigenomic research. Additionally, one genetic variant might have a different role across different types of cancer [3]. Despite the constant emergence of new technology and drugs for cancer prevention and treatment, patients with cancer still face a growing number of problems related to prevention and treatment [4]. Therefore, it would be useful to understand cancer-associated genes in cancer development through a pan-cancer analysis.

Precision medicine, a pivotal approach to managing solid-tumor patients, meticulously tailors therapeutic strategies to the unique genetic and molecular characteristics of an individual’s tumor, ensuring optimized patient outcomes. A study by elucidated the concept of utilizing next-generation sequencing (NGS) for comprehensive gene panel testing in solid tumors, highlighting the imperative nature of quality control in tissue sample handling during routine genomic testing [5]. Furthermore, research has underscored the efficacy of genomic-guided individualized precision therapy, particularly for a subset of patients navigating challenging clinical scenarios, leveraging evidence-based actionable gene variation scale tools to augment the effectiveness of genomic-guided precision therapy [6]. A pilot study demonstrated the utility of liquid biopsy in identifying actionable mutations, which are correlated with the clinical response of selected patients, showcasing the potential of this non-invasive methodology in precision medicine [7]. Moreover, the employment of a large NGS ctDNA panel via liquid biopsy has been affirmed as an efficient strategy for aligning patients with gnomically directed clinical trials and targeted therapies [8]. The MONDTI platform, through a real-world retrospective analysis, demonstrated the feasibility of precision medicine, providing a foundation for molecular-driven therapy recommendations in patients with advanced, therapy-refractory solid tumors [9]. It is also pivotal to acknowledge clonal hematopoiesis as a potential factor that could misattribute mutation origin when applying NGS findings to patient care, ensuring that results from commercial NGS assays adequately reflect the burden of somatic mutations [10]. The continuous evolution of research and clinical trials further refines and expands the application of precision medicine in the realm of oncology.

Solute carrier family 31 member 1 (*SLC31A1*) plays an important role in regulating intracellular copper homeostasis [11]; copper (Cu) is an essential micronutrient for humans. This gene encodes a high-affinity copper transporter found in the cell membrane that functions as a homotrimer to affect dietary copper uptake [12,13]. However, its role in the development of tumors is unclear. It is worth noting that a robust correlation has been observed between the expression of *SLC31A1* and the expression of PD-L1, as well as immune cell infiltration, thereby suggesting its potential significance in the context of tumor therapy [14,15,16]. Simultaneously, empirical research has demonstrated the involvement of the *SLC31A1* co-expressing gene in a substantial array of cellular processes [17]. Moreover, it was found that the overexpression of *SLC31A1* significantly increased the sensitivity of cells to physiological concentrations of copper when copper supplementation led to an overall decrease in proteins involved in mitochondrial respiration and induced cell death, which could not be reversed by inhibitors of ferroptosis, necrosis, and apoptosis [18]. Hence, we thought it was worthwhile to conduct further research on *SLC31A1*.

Our study used TCGA data to evaluate *SLC31A1* in a pan-cancer context. We examined the expression profile of *SLC31A1* and its prognostic significance among various types of cancer in humans. Furthermore, DNA methylation, immune infiltration, and protein interactions were examined. In this study, we comprehensively examined the oncogenic role of *SLC31A1* across a wide range of cancer types and demonstrated that *SLC31A1* may be an effective cancer prognostic biomarker. In summary, our data provide some insight into the increasing interest in *SLC31A1* within the context of cancer detection and therapy.

## 2. Methods

### 2.1. Gene Expression Analysis

The data obtained from the TCGA database are integrated with GTEx data to conduct an analysis of the disparities in gene expression between tumor tissues and normal tissues using the GEPIA tool. Furthermore, the expression data for *SLC31A1* in various normal cells and tissues are directly acquired from the HPA database.

#### 2.1.1. GEPIA Database

GEPIA is a web application based on the TCGA and GTEx datasets (http://gepia.cancer-pku.cn/), accessed on 1 October 2022 [19]. GEPIA serves as an interactive online platform designed for the analysis of gene expression, utilizing data derived from 9736 tumor and 8587 normal samples, sourced from the TCGA and GTEx databases. When performing research for the study that is being discussed here, we accessed the GEPIA database to evaluate the expression of *SLC31A1* in both tumor tissue and the normal tissue that corresponded to it. Then, we proceeded to present the results of our study using BodyMap and dot plots. Finally, we used this database to examine the expression of *SLC31A1* and the pathological staging of malignancies. The logarithmic scale utilized throughout was log_2_ (TPM+1). Using the “survival” module, we also examined the link between *SLC31A1* expression and the prognosis across cancers.

#### 2.1.2. HPA Database

A database called HPA (https://www.proteinatlas.org), accessed on 1 October 2022, uses antibody-based proteomics and several other holographic methods to map the whole human proteome [20]. It demonstrates the cell-type-specific spatial localization of 15,313 proteins in >40 different human tissues and organs. The HPA database served as the foundation for this investigation into the levels of *SLC31A1* mRNA expression found in various human cell lines. To represent the levels of gene expression, log TPM values were used.

### 2.2. Gene Enrichment Analysis

The GeneMANIA database is employed to derive functional assumptions for the *SLC31A1* gene, conduct gene list analysis, and ascertain the prioritization of genes for functional analysis. Concurrently, to investigate the regulatory network of *SLC31A1* in cancer, the top 10 interacting molecules of *SLC31A1* were obtained through the utilization of the STRING tool, followed by the visualization of the protein–protein interaction (PPI) network.

#### 2.2.1. GeneMANIA Database

GeneMANIA is a database for evaluating linkage data, including protein–gene relationships, pathways, co-expression, colocalization, and protein domain resemblances [21]. It can be found at http://genemania.org, accessed on 1 October 2022. In this study, we utilized this database to investigate *SLC31A1*.

#### 2.2.2. STRING Database

STRING (https://string-db.org/) can evaluate information on protein–protein interactions [22]. Our study made use of this information to examine the molecular interaction network of *SLC31A1*.

### 2.3. Genetic Alteration Analysis and DNA Methylation Analysis

The UALCAN tool is employed for the examination of DNA methylation levels in *SLC31A1* across different cancer tissues and normal tissues. Furthermore, the “TCGA Pan-Cancer Atlas Studies” module within the cBioPortal tool (http://www.cbioportal.org) was utilized to assess the genetic variations in *SLC31A1*.

#### 2.3.1. The cBioPortal Database

To investigate, display, and analyze multidimensional tumor genetic data, we checked cBioPortal (http://www.cbioportal.org), accessed on 1 October 2022 [23]. Using this database, which included a total of 2922 samples (including 2583 patients) (ICGC/TCGA, Nature 2020), we examined the pan-cancer *SLC31A1* gene mutation levels. A z-core threshold of 2.0 was used to determine the mRNA expression z-core (RNA Seq V2 RSEM).

#### 2.3.2. UALCAN Database

To examine the molecular basis of malignancies, the UALCAN database (http://ualcan.path.uab.edu/index.html), accessed on 1 October 2022, integrates genomics and bioinformatics methods [24]. The data come from the TCGA database, which has data on 33 different cancer types, including solid cancers and blood cancers, and has molecular and clinical data on more than 11,000 cancer cases. In the present investigation, we used the UALCAN database to compare the methylation levels of *SLC31A1* across cancers and their equivalents in healthy tissues. Student’s *t*-test was used to establish the statistical significance of the differences, and *p* < 0.05 was regarded as statistically significant.

### 2.4. Survival Prognosis Analysis

For the levels of *SLC31A1* expression and outcomes in patients with tumors, RNA-seq data for pan-cancer and related clinical studies were gathered from The Cancer Genome Atlas (TCGA) database. The “forestplot” R program was used to run univariate Cox regression analysis, and forest plots were utilized to display *p* values, HRs, and 95% CIs. R v4.0.3 was used to conduct statistical analyses. Unless otherwise specified, rank sum tests were used to compare two groups of data, and *p* values less than 0.05 were considered statistically significant. The Kaplan–Meier survival curve is drawn using the TIMER database (https://cistrome.shinyapps.io/timer/), accessed on 1 October 2022.

### 2.5. Immune Infiltration Analysis

For the *SLC31A1* expression, immune cell infiltration, and immunomodulatory genes, TCGA data for 33 tumors and healthy tissues were retrieved. TIMER and xCell, two methods that are combined in the R package “Immunedeconv”(v4.0.3), were used to calculate immune scores. To visualize the link between *SLC31A1* gene expression and immunological scores or immune-checkpoint-associated genes in diverse tumor types, heatmaps of Spearman correlation analysis were created, with the vertical axis indicating the immune scores and the different colors denoting correlation coefficients. For the statistical analysis, we utilized R version 4.0.3 and decided that a value of *p* < 0.05 was significant.

## 3. Results

**Figure 1:**
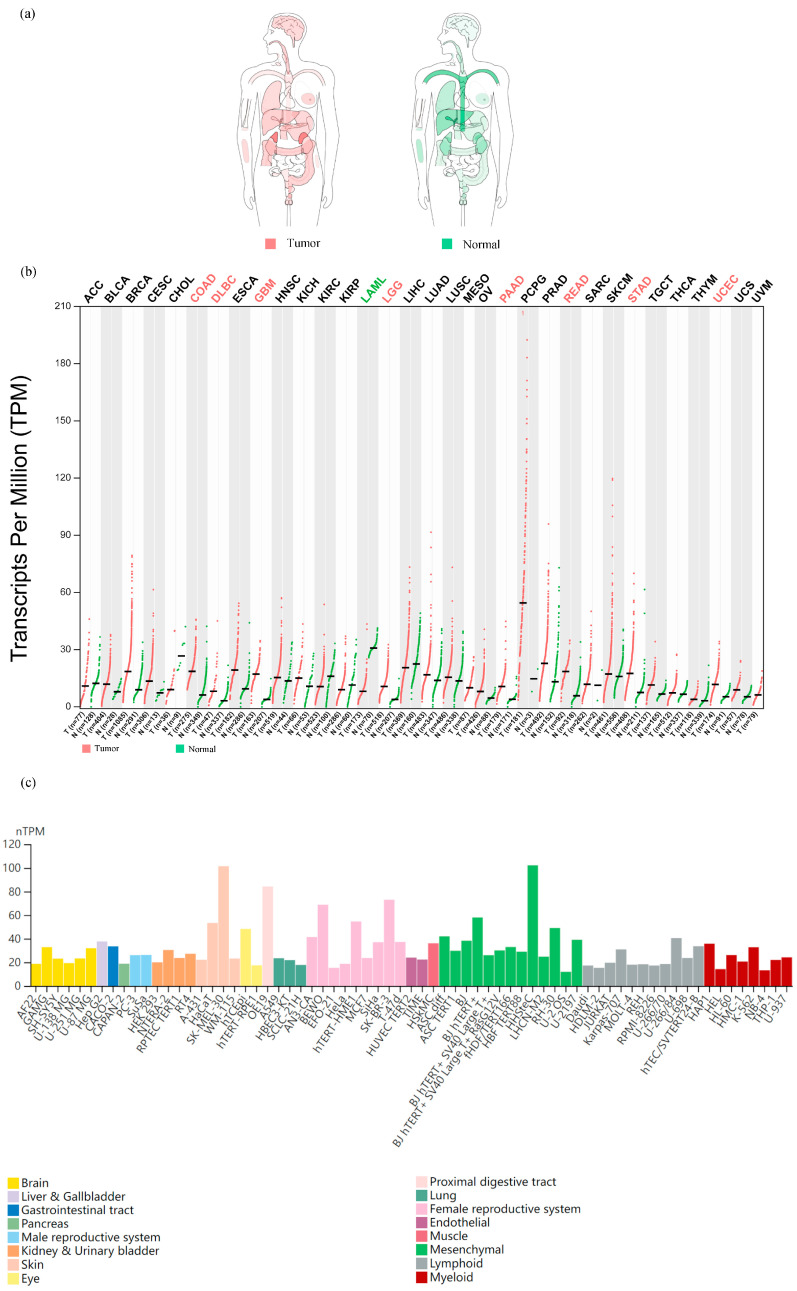
The *SLC31A1* mRNA expression landscape in humans. (**a**) Interactive BodyMap from GEPIA and a dot plot. (**b**) Showing the median *SLC31A1* expression in tumor and normal samples. Each dot represents a sample’s expression. (**c**) The HPA-database-based mRNA expression levels of *SLC31A1* in cell lines.

**Figure 2:**
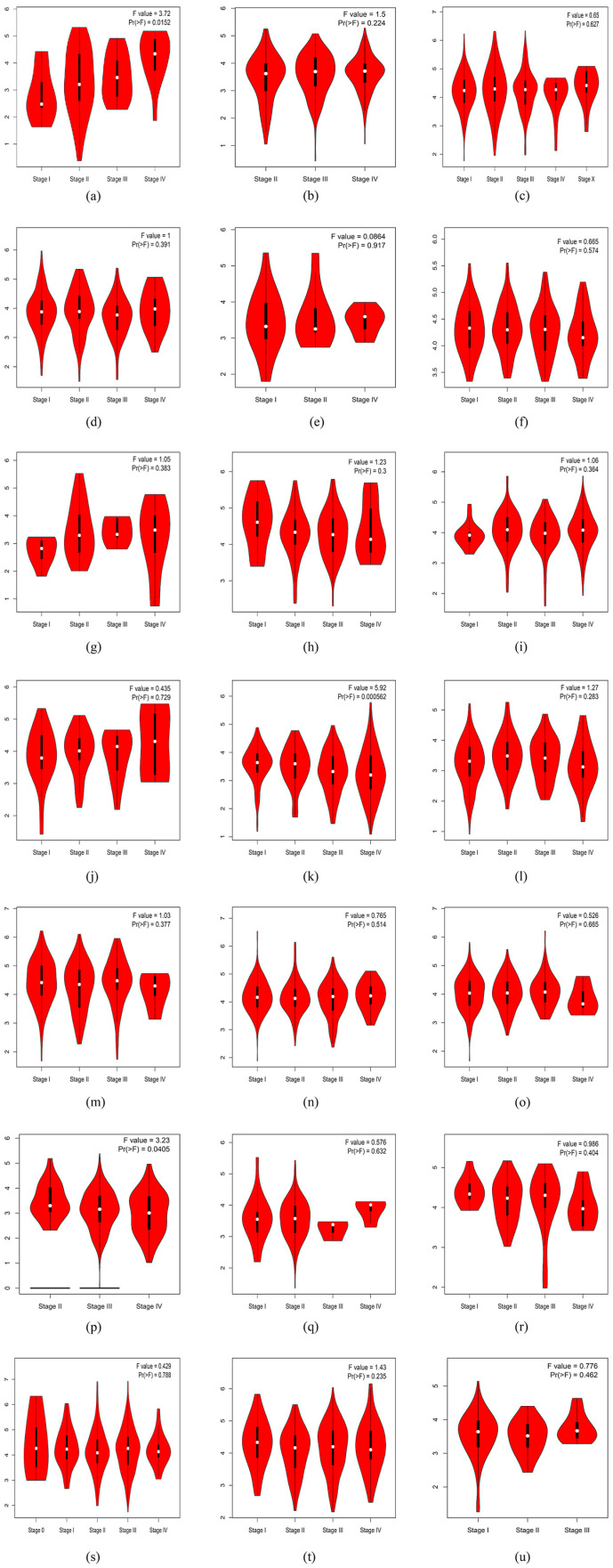

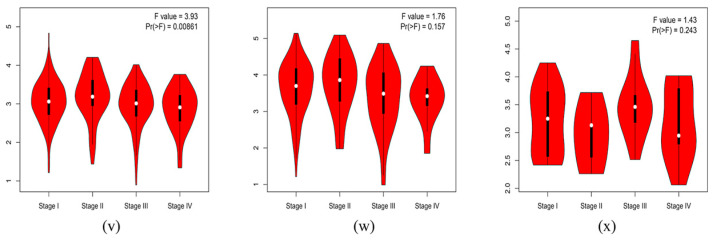
Correlations between the *SLC31A1* expression and the tumor, including the (**a**) ACC, (**k**) KIRC, (**p**) OV, and (**v**) THCA pathological stage from GEPIA. Log_2_ (TPM+1) was used for the log scale. (**a**–**x**) Represent images of SLC31A1 gene in relation to pathologic staging in ACC, BLCA, BRCA, CESC, CHOL, COAD, DLBC, ESCA, GBM, HNSC, KICH, KIRC, KIRP, LIHC, LUAD, LUSC, OV, PAAD, READ, SKCM, STAD, TGTC, THCA, UCEC, UCS.

1. A pan-cancer landscape of mRNA expression: we used the GEPIA dataset to analyze the mRNA levels of *SLC31A1* in the interactive body map to learn more about its role in human pan-cancer. *SLC31A1* expression was shown to be altered throughout many human tumor tissues compared to their corresponding normal tissues. This was notably true for the central nervous system, circulatory system, gastrointestinal system, urinary system, parathyroid glands, and thyroid (Figure 1a). Considering these results, we next examined the mRNA expression levels in 33 malignancies and adjacent normal tissues. Astonishingly, only eight tumor tissues (COAD, DLBC, GBM, LGG, PAAD, READ, STAD, and UCEC) showed higher median mRNA levels of *SLC31A1* than normal tissues (Figure 1b). Finally, we examined *SLC31A1*’s cellular mRNA expression levels using data from the HPA database. The skin, the proximal gastrointestinal tract, the female reproductive system, the eye, and mesenchyme were among the tissue organ cell lines with higher *SLC31A1* mRNA expression levels (Figure 1c).
2. *SLC31A1* expression and the pathological staging of cancers have been shown to have a substantial relationship. The pathological staging of malignancies is one of the key indications of patient prospects. As a result, our research investigated the connection between the *SLC31A1* expression levels in cancers and their pathological stages using GEPIA, and it included 17 different malignancies. It is interesting to note that the level of *SLC31A1* expression was not found to relate to the pathological stage of any other tumors, apart from ACC (*p* = 0.0152), KIRC (*p* = 0.000562), OV (*p* = 0.0405), and THCA (*p* = 0.00861); *SLC31A1* exhibited an upward trend in relation to the pathological stage in ACC, while displaying a contrasting pattern in KIRC, OV, and THCA (Figure 2). The level of expression of *SLC31A1* was shown to be linked with the pathological staging of ACC, KIRC, OV, and THCA, which suggests that it may be of importance in guiding the pathological staging of these malignancies. Interestingly, additional analyses conducted on the identical open-source database produced congruent experimental outcomes to ours, thereby providing further validation of the dependability of our results [17].

**Figure 3:**
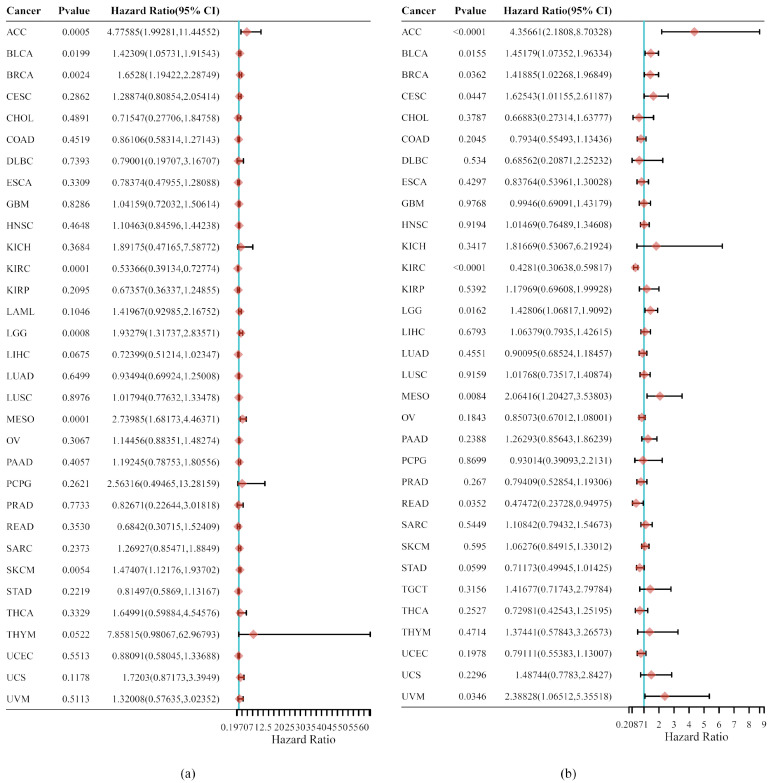
Cancer patients’ prognosis and *SLC31A1* expression are related. The hazard ratios of *SLC31A1* in 33 different types of tumors are plotted in a forest in (**a**,**b**).

Insights into the relationship between *SLC31A1* expression and tumor outcome: Cox regression analysis was used to perform more research into the survival prospects of pan-cancer patients, taking into consideration the amount of *SLC31A1* expression in the tumor, as well as the pathological stage of the tumor. Notable prognostic factors mostly included overall survival and progression-free survival in this investigation. *SLC31A1* expression levels were shown to have a significant correlation with overall survival in patients suffering from ACC, BLCA, BRCA, KIRC, LGG, MESO, and SKCM, as determined via a Cox regression study of 33 different forms of tumors (Figure 3a). In addition, we discovered that *SLC31A1* expression has a significant correlation with PFS in eight different cancers, including ACC, BLCA, BRCA, CESC, KIRC, LGG, MESO, and UVM (Figure 3b). These tumors were tested. After analyzing the data with Kaplan–Meier survival curves, we concluded that a high expression of *SLC31A1* in ACC, BLCA, BRCA, LGG, MESO, SKCM, THYM, UCS, TGCT. (Supplementary Figure S1) was associated with worse overall survival (OS), whereas a low expression of *SLC31A1* in KIRC (Supplementary Figure S2) was associated with a worse overall survival (OS). Detailed information on the relationship of other tumor species to the OS can be found in the supplementary material (Supplementary Figure S3).

**Figure 4:**
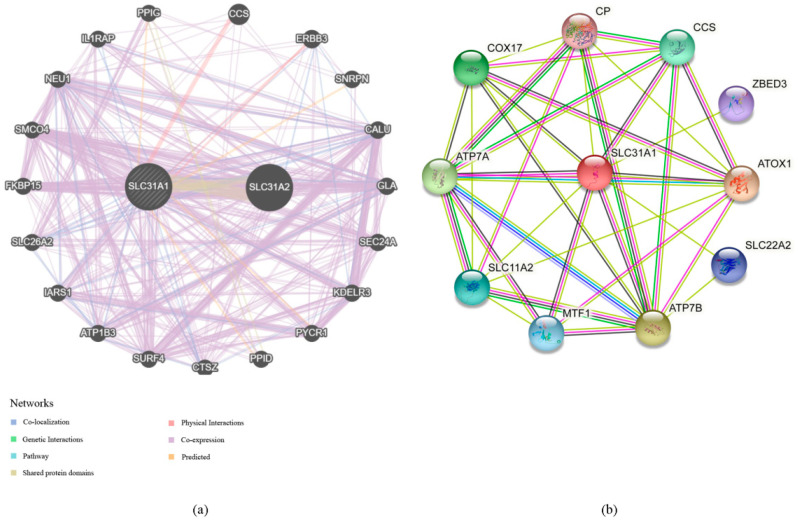
The network of protein–protein interactions and functional enrichment of the *SLC31A1* gene. (**a**,**b**) Protein–protein interaction network using the GeneMANIA and STRING databases.

**Figure 5:**
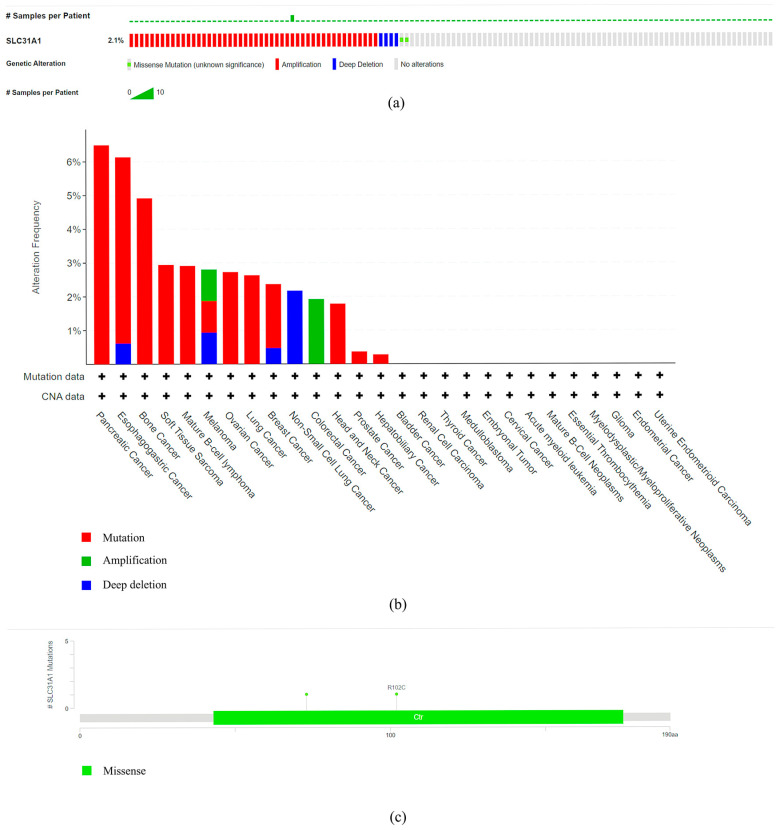
Analysis of the pan-cancer *SLC31A1* gene mutation level. (**a**) A genome-wide pan-cancer study in the cBioPortal database (ICGC/TCGA, Nature 2020) was used to evaluate the number of mutations in the *SLC31A1* gene. (**b**) The cBioPortal database was used to study how often various mutations were found in the *SLC31A1* gene. (**c**) A protein domain-by-domain representation of *SLC31A1* mutations in various cancers.

**Figure 6:**
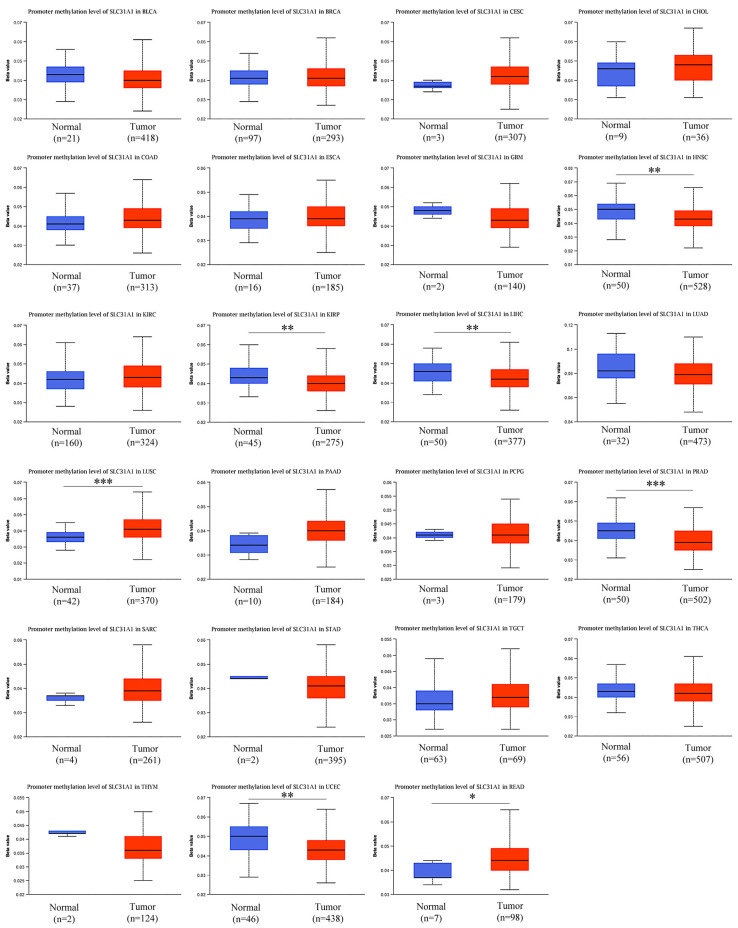
Analysis of pan-cancer *SLC31A1* methylation levels. The use of the UALCAN database for an examination of pan-cancer *SLC31A1* methylation (* *p* < 0.05, ** *p* < 0.01, and *** *p* < 0.001).

**Figure 7:**
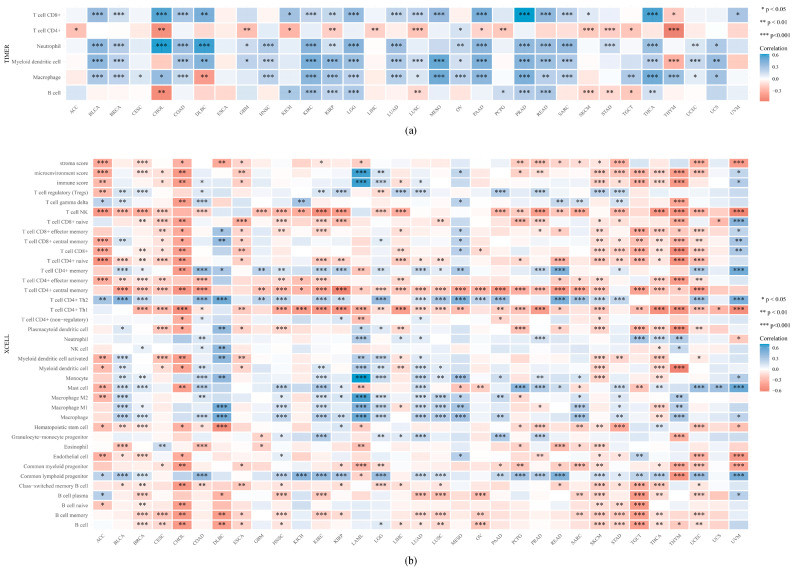
A comprehensive investigation of the expression of *SLC31A1* and the presence of immune cell infiltration. (**a**) In the TIMER database, the *SLC31A1* expression was strongly linked to the number of immune cells that got into the body. (**b**) According to xCell, the level of *SLC31A1* expression was strongly related to the number of immune cells that got in (* *p* < 0.05, ** *p* < 0.01, and *** *p* < 0.001).

3. Our investigation into the GeneMANIA databases led us to the discovery of 20 genes that are linked with the protein–protein interactions of *SLC31A1* (Figure 4a). The small molecule route and protein–protein interaction network of *SLC31A1* are shown below. According to the information found in the STRING database, there are a total of 10 nodes connected to the *SLC31A1* gene (Figure 4b).
4. An investigation into the mutations of the *SLC31A1* gene and the methylation levels of pan-cancerous tumors: the cBioPortal database was analyzed, and the results showed that 2.1% (54 out of 2584) of pan-cancer patients had mutations in the *SLC31A1* gene (Figure 5a). In addition, we investigated the prevalence of mutations in the *SLC31A1* gene among the various tumor types. The results showed that the disease with the highest frequency of aberrations was pancreatic cancer, followed by esophageal and gastric cancer and bone cancer (Figure 5b). Notably, mutations are the most common *SLC31A1* aberrations. Our research found a total of two mutation sites, both of which were situated between numbers 0 and 200 (Figure 5c). This was done so that we could learn more about the SLC31A mutation sites found throughout the protein domains involved in cancer.
5. DNA that has been methylated incorrectly is a substantial contributor to the development of cancer. Therefore, in the next step, we analyzed *SLC31A1* methylation across cancers and the tissues that correlate with it using the UALCAN database. Compared to normal tissues, the levels of *SLC31A1* methylation in HNSC, KIIRP, LIHC, LUSC, PRAD, READ, and UCEC tissues were found to be very different (Figure 6).

6. The expression and permeation of immunocytes in pan-cancers: in terms of the reality that there is a connection between *SLC31A1* and the immune response, we decided to carry out pan-cancer research to investigate the link between *SLC31A1* and the degree to which immune cells infiltrated the cancerous tissue. According to the data available here, 20 tumors were related to CD8+ T cells, 14 tumors were related to CD4+ T cells, 20 tumors were related to neutrophils, 21 tumors were related to medullary dendritic cells, 23 tumors were related to macrophages, and 13 tumors were related to B cells (Figure 7a).

To further determine the correlation of the expression of *SLC31A1* with different types of immune cell invasion, we explored this using the xCell online tool. Among the subtypes of immune cells, the expression of *SLC31A1* was negatively associated with those of ACC, CESC, CHOL, ESCA, SKCM, STAD, TGCT, THCA, and THYM, while there was a substantial positive correlation between the expression levels of *SLC31A1* and those of DLBC, LAML, and MESO. It is worth noting that NK T cells, CD8+ naïve T cells, CD4+ central memory T cells, CD4+ Th1 T cells, memory B cells, and *SLC31A1* had the most powerful negative correlations among multiple cancers (Figure 7b).

**Figure 8:**
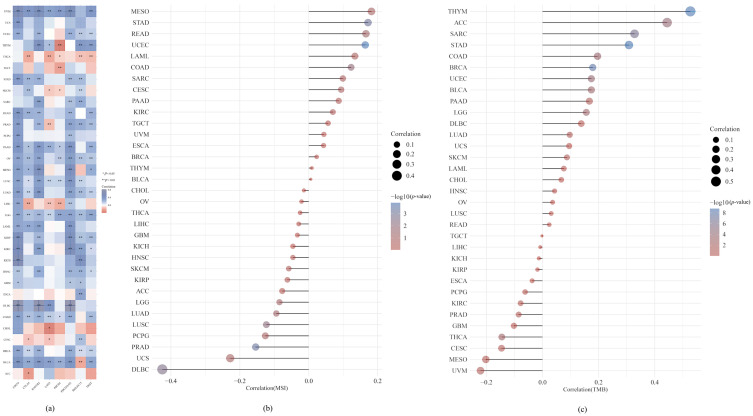
*SLC31A1* expression, immune checkpoint genes, and the immunological regulators TMB and MSI were analyzed throughout the spectrum of cancers. (**a**) A global examination of the relationship between immune checkpoint genes and *SLC31A1* expression. (**b**) A global examination of the relationship between the immunomodulator TMB and *SLC31A1* expression. (**c**) Analyzing the connection between the immunomodulator MSI and *SLC31A1* expression on a global scale (* *p* < 0.05, ** *p* < 0.01).

7. We performed an analysis of the expression of *SLC31A1* across many cancer types, together with the immune regulators TMB and MSI, and the immunological checkpoints. We assessed the link between *SLC31A1* expression and two important immune regulators to quantify the relationship between *SLC31A1* expression and the TME in the pan-cancer dataset. This allowed us to better understand the nature of this interaction. Positive associations were found between immune checkpoint genes and most different types of cancer, including UVM, UCEC, STAD, READ, OV, PAAD, LGG, LUSC, LAML, LUAD, DLBC, COAD, and BLCA. Only a small percentage of cancers, including THCA and CHOL tumors, were shown to have a negative association with immune checkpoint genes (Figure 8a).

In past studies, TMB and MSI were shown to be indications of the patient response to medicine, especially for immune checkpoint inhibitors that attempt to block PD-1/PD-L1 or CTLA4 [25]. First, we investigated the possible relationship between TMB and the expression of *SLC31A1*. According to the findings of our study, the levels of *SLC31A1* expression are significantly correlated with TMB in several cancers, including THYM, ACC, SARC, and STAD (Figure 8b). In this paper, we also found that the levels of *SLC31A1* expression were closely linked to MSI in a wide range of cancers, including STAD and UCEC (Figure 8c).

## 4. Discussion

Solute carrier family 31 member 1 (*SLC31A1*) is a homotrimer that plays a crucial role in copper homeostasis by regulating dietary copper intake [11]. This is not all that *SLC31A1* affects; it also plays a role in the formation of tumors, among other things. Recent research suggests that *SLC31A1*, as a member of the cuproptosis family, is closely linked to the onset, development, and outcome of cancer [26,27,28]. Therefore, the significance of *SLC31A1* in human cancers was studied using bioinformatics. First, we compared the levels of *SLC31A1* expression between several human malignancies and their corresponding normal tissues using the database BodyMap. We further show that *SLC31A1* expression is associated with the pathological staging of ACC, KIRC, OV, and THCA, as well as the prognosis of seven cancers, including ACC, BLCA, BRCA, KIRC, LGG, MESO, and SKCM. We also discovered that *SLC31A1* methylation levels were much lower in the vast majority of malignant tumors. Furthermore, *SLC31A1* was linked to immune cell infiltration in the cancer microenvironment.

Our findings suggest that malignant tissues, including tumors of the brain, lymph nodes, gastrointestinal tract, and genitourinary system, express more *SLC31A1* mRNA than their healthy counterparts. Due to the brain’s advanced function as a nerve center, normal brain functions require high amounts of metal [29]. The brain is, therefore, very responsive to variations in copper levels [30]. *SLC31A1* is required for development, iron metabolism, and proper heart function in newborns, and it is the principal mechanism promoting intestinal Cu absorption in animals [31]. *SLC31A1* is important not only in normal tissues but also in the development and treatment of a variety of tumors. It was shown that in human ovarian tumors, low levels of *SLC31A1* mRNA were associated with adverse clinical responses to platinum-based therapies, while a copper chelator enhanced the ability of cisplatin to kill cultured human ovarian cancer cells. In a mouse model of human cervical cancer, we demonstrated that combination therapy with a copper chelator and cisplatin increased the levels of cisplatin–DNA adducts in tumor tissues and improved the therapeutic efficacy [32]. Without *SLC31A1*, lung tumors that were driven by KRASG12D grew and lived for a shorter period. This was linked to lower levels of autophagy and signal transduction [33].

In addition, we found that *SLC31A1* expression levels were significantly correlated with ACC, KIRC, OV, and THCA pathological stages only, not with other tumor stages. According to our results, *SLC31A1* may serve as a valuable new pathological staging marker for patients with ACC, KIRC, OV, and THCA. However, it is unclear why *SLC31A1* is solely linked to pathological staging in LIHC; therefore, this is an area that needs more investigation. In contrast, we show that *SLC31A1* expression levels are connected to ACC, BLCA, BRCA, KIRC, LGG, MESO, and SKCM survival outcomes in pancreatic cancer. How does *SLC31A1* influence the prognosis of cancer patients? *SLC31A1* affects oncogenic BRAF signaling and tumorigenesis by regulating intracellular copper levels [34]. Additionally, its role in transporting platinum-based chemotherapeutic agents, regulating PD-L1 expression, and influencing tumor immune escape cannot be ignored [14]. Interactions between copper transport proteins and cellular senescence and impaired autophagy have also been reported [28,35]. All these could be potential solutions to this question.

We found 10 genes connected to *SLC31A1* by analyzing protein–protein interaction networks. All the genes connected to *SLC31A1* have been implicated in cancer. These data suggest that COX17 function upregulation and increased cytochrome c oxidase activity are common features of lung carcinogenesis [36]. In addition, a study showed that Atox1 mediates breast cancer cell migration through synergistic copper transport on the ATP7A–LOX axis and may be a predictor of metastatic potential [37,38]. Because of this, we now have knowledge about diagnosing and treating pan-cancer that we did not have before.

During cancer development and growth, genetic errors are revealed. There are many additional “passengers” found in cancer genomes. In cancer research, mutations known as “drivers” have been identified and reveal basic biological processes that are not functioning properly and lead to cancer [39]. As part of precision oncology, these drivers are the target of new therapies that allow patients to be treated based on the genetic changes present in their tumors. Interestingly, our data suggest that *SLC31A1* is mutated at a rate of 2.1% in pan-cancer cells. Therefore, several studies have developed hereditary loss-of-function mutation models of copper delivery genes to study individual changes in restoring intracellular copper homeostatic drug action [40]. Indeed, DNA methylation analysis is a promising tool that can improve early diagnosis accuracy by detecting altered DNA methylation in circulating tumor DNA [41,42,43]. DNA methylation presents us with a promising future for minimally invasive cancer detection and classification [44]. Based on the UALCAN database, we provide preliminary evidence that seven tumors are methylated less frequently than the norm. A study confirmed that reduced levels of DNA methylation predispose cells to the activation of gene transcription, thereby increasing the ability of tumors to proliferate, migrate, and metastasize [45,46,47]. Therefore, DNA hypomethylation predicts a poor prognosis for these tumors [48,49].

We conducted an analysis of the expression of *SLC31A1* across many cancer types, together with the immune regulators TMB and MSI, and the immunological checkpoints. We assessed the link between *SLC31A1* expression and two important immune regulators to quantify the relationship between *SLC31A1* expression and the TME in the pan-cancer dataset. This allowed us to better understand the nature of this interaction. Positive associations were found between immune checkpoint genes and many different types of cancer, including UVM, UCEC, STAD, READ, OV, PAAD, LGG, LUSC, LAML, LUAD, DLBC, COAD, and BLCA. Only a small percentage of cancers, including THCA and CHOL tumors, were shown to have a negative association with immune checkpoint genes.

In past studies, TMB and MSI were shown to be indications of the patient response to medicine, especially for immune checkpoint inhibitors that attempt to block PD-1/PD-L1 or CTLA4 [25,50]. First, we investigated the possible relationship between TMB and the expression of *SLC31A1*. According to the findings of our study, the levels of *SLC31A1* expression are significantly correlated with TMB in a number of cancers, including THYM, ACC, SARC, and STAD. Additionally, in this paper, we concluded that the levels of *SLC31A1* expression were closely associated with MSI in a variety of malignancies, including STAD and UCEC.

Considering the results of these investigations, further study into the link between *SLC31A1* and immunomodulators was performed. The findings revealed that the majority of malignancies were found to have a positive correlation with immune checkpoint genes. Only a few cancer types, including THCA and CHOL, were shown to have a negative correlation with immune checkpoint genes. Furthermore, we analyzed the correlation among *SLC31A1* and TMB and MSI and found that the *SLC31A1* expression levels in THYM, ACC, SARC, and STAD were substantially connected with TMB and that *SLC31A1* expression levels in STAD and UCEC were correlated with MSI. This was the result of our investigation. Nevertheless, sadly, no other researchers have further studied the deeper relationship between *SLC31A1* and either of the immunomodulators. This is something that we would like to see change in the future.

Significantly, discrepancies in *SLC31A1* expression have been documented in various cancers. For instance, when contrasted with normal tissue, the gene expression of *SLC31A1* in ACC does not show an elevation, yet it intensifies with the progression of the disease. It is universally acknowledged that genes do not function in isolation: the expression of the proteins they encode is modulated by a myriad of factors, including, but not limited to, other genes, environmental influences, and epigenetic changes [51]. Importantly, mRNA transcription does not always correspond to protein expression, and variances between mRNA and protein levels are frequently observed [52]. The divergent expression of *SLC31A1* at both the mRNA and protein levels within identical cancer types suggests that delving into the regulatory mechanism of *SLC31A1* expression is of paramount importance. The distinct role of *SLC31A1* in various cancers hinges on the specific cancer subtype, and both gain-of-function and loss-of-function experiments are likely to elucidate its definitive role. Additionally, when integrated with survival analysis data, these findings highlight the prognostic significance of *SLC31A1* across a spectrum of cancer variants. While promoter methylation is generally associated with gene silencing [53], there are instances where genes can be hypomethylated and overexpressed, leading to advanced cancer stages and poor prognoses [54]. The exact mechanisms behind these phenomena can be multifactorial, involving various epigenetic, genetic, and environmental factors [55]. The correlation between a specific gene and TMB/MSI and immune cell infiltration can vary across different tumor types due to the complex interplay of genetic, epigenetic, and environmental factors [56,57]. The tumor microenvironment, the specific type of immune cells present, and the overall genetic landscape of the tumor can all influence how a particular gene behaves in the context of cancer [57,58].

## 5. Conclusions

According to the findings of this research, members of the solute carrier family 31 member 1 (*SLC31A1*) pan-cancer share certain traits but also have some distinct variations. When viewed as a whole, our findings suggest that the levels of *SLC31A1* gene expression in various cancers display a considerable amount of variety. Consequently, more research focusing on forms of cancer is required. In general, the findings of our research indicate that this gene (*SLC31A1*) has a function in expression, prognosis, DNA methylation, immune cell infiltration, the expression of immune checkpoint genes, and immunotherapeutic indicators (TMB and MSI). This research will evaluate concepts that have previously been proposed and provide fresh insight into the investigation of the processes underpinning *SLC31A1* in 33 distinct forms of cancer.

## Data Availability

The datasets analyzed during the current study are available in the GEPIA database (http://gepia.cancer-pku.cn/), HPA database: (https://www.proteinatlas.org), GeneMANIA database (http://genemania.org), STRING database (https://string-db.org/), cBioPortal database (http://www.cbioportal.org), UALCAN database (http://ualcan.path.uab.edu/index.html), TCGA database (https://tcga-data.nci.nih.gov/tcga), or TIMER database (https://cistrome.shinyapps.io/timer/).

## Supplementary Materials

The following supporting information can be downloaded at: https://www.mdpi.com/article/10.3390/biomedicines11112884/s1.

## Abbreviations

*SLC31A1*	Solute carrier family 31 member 1
ACC	Adrenocortical carcinoma
BLCA	Bladder urothelial carcinoma
BRCA	Breast invasive carcinoma
CESC	Cervical squamous cell carcinoma
CHOL	Cholangiocarcinoma
COAD	Colon adenocarcinoma
DLBC	Lymphoid neoplasm diffuse large B cell lymphoma
ESCA	Esophageal carcinoma
GBM	Glioblastoma
LGG	Brain lower grade glioma
HNSC	Head and neck squamous cell carcinoma
KICH	Kidney chromophobe
KIRC	Kidney renal clear cell carcinoma
KIRP	Kidney renal papillary cell carcinoma
LAML	Acute myeloid leukemia
LIHC	Liver hepatocellular carcinoma
LUAD	Lung adenocarcinoma
LUSC	Lung squamous cell carcinoma
MESO	Mesothelioma
OV	Ovarian serous cystadenocarcinoma
PAAD	Pancreatic adenocarcinoma
PCPG	Pheochromocytoma and paraganglioma
PRAD	Prostate adenocarcinoma
READ	Rectum adenocarcinoma
SARC	Sarcoma
SKCM	Skin cutaneous melanoma
STAD	Stomach adenocarcinoma
TGCT	Testicular germ cell tumors
THCA	Thyroid carcinoma
THYM	Thymoma
UCEC	Uterine corpus endometrial carcinoma
UCS	Uterine carcinosarcoma
UVM	Uveal melanoma
COX17	Cytochrome c oxidase copper chaperone
ATOX1	Antioxidant 1
