# Supplementary material for: SLC31A1 Identifying a Novel Biomarker with Potential Prognostic and Immunotherapeutic Potential in Pan-Cancer
Source: Biomedicines. 2023 Oct 25;11(11):2884. doi: 10.3390/biomedicines11112884 (PMC10669416; doi:10.3390/biomedicines11112884)
Supplement: Supplementary file 1 [file biomedicines-11-02884-s001.zip › biomedicines-2641945-supplementary.pdf]

Supplementary Material

1.1 Supplementary Figures

Figure S1

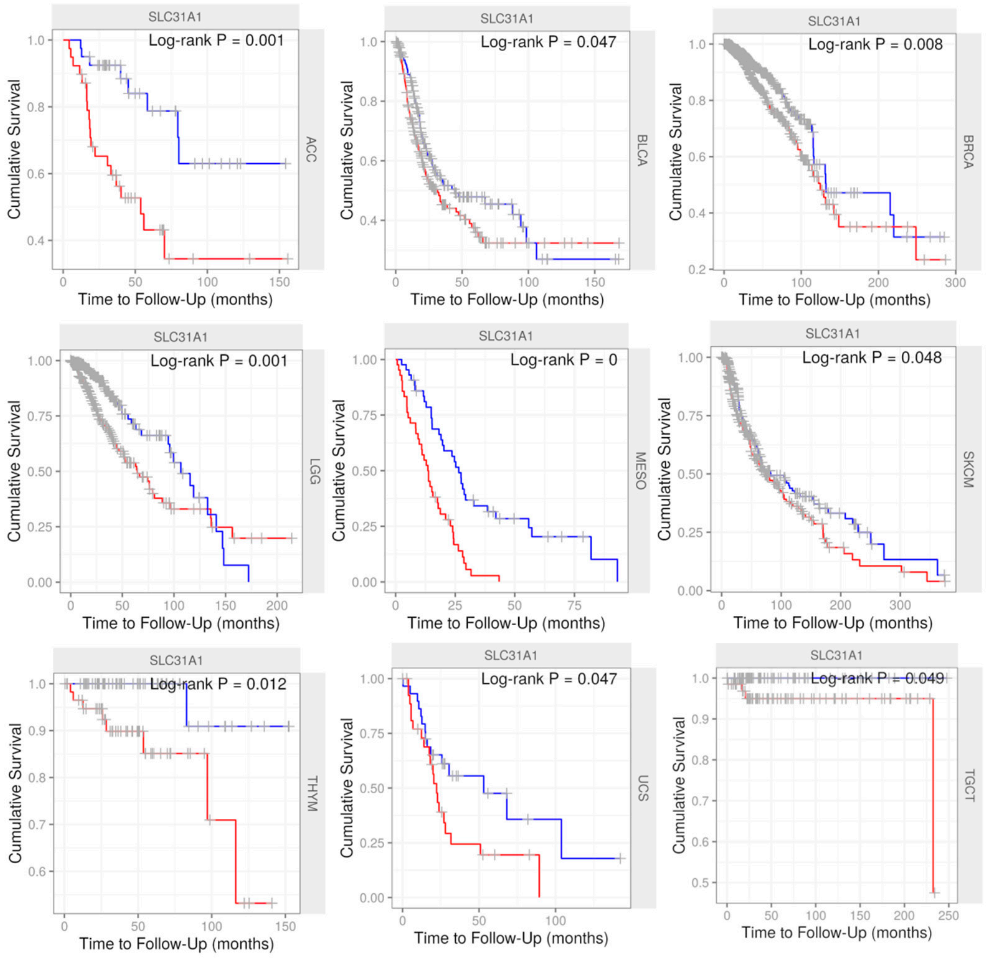

Supplementary Figure S1. Expression of and overall survival in cancer patients. OS Kaplan-Meier survival curves stratified by the varied expressions in ACC, BLCA, BRCA, LGG, MESO, SKCM, THYM, UCS, TGCT.

Figure S2

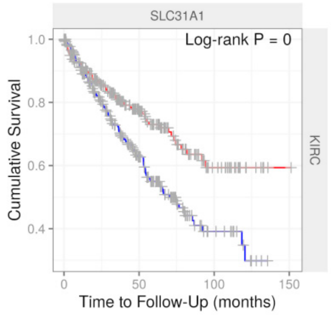

Supplementary Figure S2. Expression of and overall survival in cancer patients. OS Kaplan-Meier survival curves stratified by the varied expressions in KIRC.

Figure S3

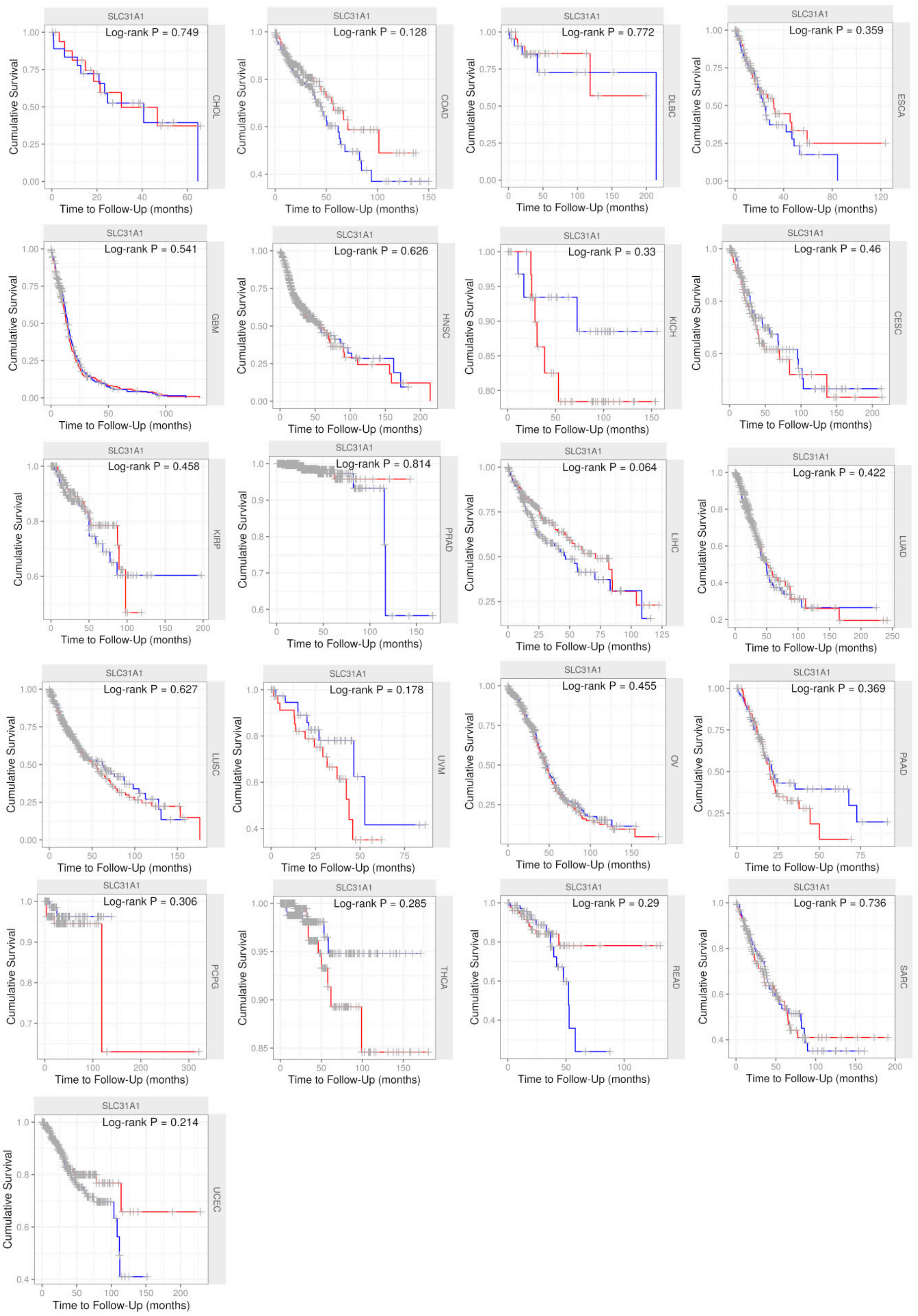

SLC31A1
Log-rank P = 0.749
CHOL
SLC31A1
Log-rank P = 0.128
COAD
SLC31A1
Log-rank P = 0.772
DLBC
SLC31A1
Log-rank P = 0.359
ESCA
SLC31A1
Log-rank P = 0.541
GBM
SLC31A1
Log-rank P = 0.626
HNSC
SLC31A1
Log-rank P = 0.33
KICH
SLC31A1
Log-rank P = 0.46
CESC
SLC31A1
Log-rank P = 0.458
KIRP
SLC31A1
Log-rank P = 0.814
PRAD
SLC31A1
Log-rank P = 0.064
LIHC
SLC31A1
Log-rank P = 0.422
LUAD
SLC31A1
Log-rank P = 0.627
LUSC
SLC31A1
Log-rank P = 0.178
UVM
SLC31A1
Log-rank P = 0.455
OV
SLC31A1
Log-rank P = 0.369
PAAD
SLC31A1
Log-rank P = 0.306
PCPG
SLC31A1
Log-rank P = 0.285
THCA
SLC31A1
Log-rank P = 0.29
READ
SLC31A1
Log-rank P = 0.736
SARC
SLC31A1
Log-rank P = 0.214
UCEC
Cumulative Survival
Time to Follow-Up (months)

Supplementary Figure S3. Expression of and overall survival in cancer patients. OS Kaplan-Meier survival curves stratified by the varied expressions in CHOL, COAD, DLBC, ESCA, GBM, HNSC, CESC, KICH, KIRP, LIHC, LUAD, LUSC, UVM, OV, PAAD, PCPG, PRAD, THCA, READ, SARC, UCEC.
